# Healthcare Professionals’ Cultural Competence in Diabetes Care: A Systematic Review

**DOI:** 10.3390/healthcare13222910

**Published:** 2025-11-14

**Authors:** Monica Nikitara, Achonwa Esther Mba, Evangelos Latzourakis, Costas S. Constantinou

**Affiliations:** 1 Department of Health Sciences, School of Life and Health Sciences, University of Nicosia, Nicosia 2417, Cyprus; 2 Department of Basic and Clinical Sciences, Medical School, University of Nicosia, Nicosia 2417, Cyprus

**Keywords:** cultural competence, Diabetes Mellitus, healthcare providers

## Abstract

Background: Culturally diverse patients with diabetes often face barriers that contribute to poor outcomes. Providing culturally sensitive care requires awareness of how cultural beliefs influence management, yet no standard model of cultural competency exists, underscoring the need for further research. Aims: To evaluate the level of cultural competence among healthcare professionals in caring for patients with diabetes, and to assess the impact of cultural competence training on their ability to deliver culturally sensitive, patient-centered care. Methodology: A systematic review was conducted of primary research articles published between 2015 and 2025 that examined the cultural competence of healthcare providers in diabetes care, described relevant training programs, and evaluated their impact. The databases searched included Medline, CINAHL, ProQuest, and the Nursing and Allied Health Database. Result: A total of 15 studies were included in the review. Seven assessed the cultural competence of diabetes care providers, reporting moderate to high levels of awareness and sensitivity but noting gaps in communication and cultural knowledge. Eight studies evaluated training interventions, all of which demonstrated improvements in provider attitudes and self-perceived competence. Some also reported better patient outcomes, particularly among high-risk groups. However, the long-term effects were inconsistent, and no single assessment tool proved universally effective. Conclusion: This systematic review suggests that the cultural competence of healthcare providers in diabetes care remains limited, although some evidence indicates that interventions can enhance competence. The findings may assist researchers in selecting appropriate measures to evaluate cultural competence in diabetes care.

## 1. Introduction

Diabetes Mellitus is a rapidly growing global health issue, currently affecting an estimated 830 million individuals [1]. In 2021 alone, the disease accounted for over 2 million deaths and contributed to 11% of cardiovascular mortality worldwide [2]. As a progressive condition, type 2 diabetes can lead to both microvascular and macrovascular complications. However, these complications can often be prevented, controlled, or delayed through effective self-care [3]. Active engagement in self-care, grounded in a clear understanding of illness, is therefore central to effective management. Self-care practices are shaped by multiple factors, including knowledge of the condition, cultural beliefs and values, prior experiences, social support, access to resources, skills, motivation, habits, and functional or cognitive abilities. Because individuals’ responses to healthcare guidance are partly shaped by their cultural background, integrating cultural values into diabetes care is essential [4].

The number of international migrants has increased substantially over the past five decades. According to the International Organization for Migration (IOM), there were approximately 281 million international migrants worldwide in 2020, representing 3.6% of the global population. Migration flows reached unprecedented levels in 2022, with over 6 million new permanent immigrants (excluding 4.7 million Ukrainian refugees), driven by factors such as labor shortages and the COVID-19 pandemic [5]. These migratory patterns have heightened cultural and ethnic diversity in host countries, underscoring the need for culturally competent healthcare to address the varied health needs of migrant populations [6]. Cultural competence is the ability to recognize how culture shapes beliefs and behaviors and to adapt care to improve outcomes. Its key components are awareness of one’s own biases, knowledge of cultural groups, sensitivity to differences, and the skills to communicate effectively across cultures [7].

In this context, effective cross-cultural communication emerges as a cornerstone of culturally competent care, particularly in the management of chronic conditions such as diabetes. Effective communication between healthcare providers and patients is essential, as provider responses during consultations influence patient understanding and engagement. This is particularly critical in type 2 diabetes care, which disproportionately affects vulnerable populations, including migrants in high-income countries. Diabetes care is marked by significant ethnic and racial disparities. In the U.S., Hispanics and non-Hispanic Black populations are less likely to meet diabetes control targets or receive preventive care, leading to higher rates of complications [8]. Migrants with type 2 diabetes also show limited awareness of the disease and often feel that care does not meet their cultural needs [7]. Consequently, these groups face higher risks of complications such as lower-limb amputations, retinopathy, and end-stage renal disease. The reasons for these disparities are multifactorial; they include access to care and healthcare delivery, and social, biological, and lifestyle factors [9].

Healthcare providers play a key role in reducing racial and ethnic disparities in diabetes care, yet many face barriers such as language and cultural differences that threaten patient safety [9]. Limited awareness of health inequalities, poor recognition of sociocultural factors, and fear of cross-cultural interactions can hinder communication and reinforce stereotypes [10,11,12]. Enhancing awareness and reflection on sociocultural influences is therefore critical for improving provider–patient interactions. Culturally competent care addresses the diverse needs of patients and has become a global priority [13]. To sustain competence, training should be continuous, context-specific, and developed with input from stakeholders, including patients. While evidence on patient outcomes remains limited, such training consistently improves healthcare professionals’ knowledge, attitudes, and behaviors. Further research is needed to identify the most effective and feasible educational models and resource requirements. Therefore, this review aims to evaluate the level of cultural competence among healthcare professionals in caring for patients with diabetes, and to assess the impact of cultural competence training on their ability to deliver culturally sensitive, patient-centered care. A systematic review approach was adopted to enable the synthesis and critical appraisal of existing empirical evidence on this topic [14].

## 2. Materials and Methods

This systematic review was conducted in accordance with PRISMA guidelines 2020 to comprehensively identify, appraise, and synthesize relevant evidence [15]. Prospero Registration Number: CRD420251150622.

### 2.1. Eligibility Criteria

Table 1 summarizes the inclusion and exclusion criteria applied to choose the articles.

### 2.2. Information Sources and Search Strategies

We conducted a comprehensive search across MEDLINE, CINAHL, ProQuest, and the Nursing & Allied Health Database to cover nursing and allied health literature relevant to cultural competence.

The search combined keywords with controlled vocabulary terms such as MeSH and CINAHL Subject Headings for “cultural competence,” “healthcare professionals,” “diabetes,” and “training or education.” Boolean operators were applied: OR within each concept and AND between the main concepts. The database-specific EBSCOhost search strings are shown in Table 2.

The search was restricted to English-language, peer-reviewed, full-text journal articles. Records unrelated to healthcare professionals or diabetes were excluded.

### 2.3. Search Keyword and Strategy

The PIC (Population, Interest, Context) framework (Table 2) was used to structure the research questions and to develop effective search strategies.

### 2.4. Study Selection Process

Two researchers independently searched, screened, and selected studies using three primary criteria: (a) primary research, (b) a focus on diabetes, and (c) involvement of healthcare professionals. Additional refinements (publication date and peer-review status) were then applied. Eligible records were collated, and the final set of studies for inclusion was agreed upon.

### 2.5. Data Collection Process

Two researchers independently extracted data from the included studies, noting the competencies identified (and agreed upon by experts within each study). They collected the study title, authors, year of publication, aim, methodology/design, and a summary of results and findings. Data were analyzed iteratively, and overarching themes were subsequently identified.

### 2.6. Data Items

We extracted study title, authors, year, country/setting, publication type, and design; sample size and participant characteristics (profession/discipline, years in practice/prior cultural training, and age/sex where reported). For interventions, we recorded training content/pedagogy, mode, duration, and comparators. Outcomes included provider cultural-competence measures (instrument/subscales and baseline/post/follow-up scores), consultation behaviors (e.g., teach-back), patient outcomes (e.g., HbA1c, satisfaction), and service-level changes (e.g., interpreters, tailored materials). We also noted reported barriers/facilitators, follow-up length, and funding/conflicts of interest.

### 2.7. Study Risk of Bias Assessment

Joanna Briggs Institute (JBI) Critical Appraisal Checklists was used to evaluate the risk of bias in the included studies. These assess the methodological quality of research and detect possible biases in the analysis, design, and execution.

### 2.8. Effect Measures

The outcomes of interest were: (1) health providers’ levels of cultural competence and (2) the barriers faced by professionals when caring for diverse patients with diabetes. These were assessed in studies using interviews, questionnaires, and validated scales. A further outcome was the impact of training on clinicians’ knowledge and skills, evaluated with standardized instruments and qualitative analyses.

### 2.9. Synthesis Methods

Given the heterogeneity of study designs and outcomes, a convergent mixed-methods synthesis approach was used. Quantitative results (e.g., pre/post intervention scores, HbA1c levels, and competence scales) were summarized descriptively and compared narratively across studies. Qualitative findings (e.g., perceptions of cultural competence, barriers to implementation) were analyzed using qualitative content analysis to identify recurring themes and patterns. The findings from quantitative and qualitative strands were then integrated narratively, highlighting points of convergence and divergence. Meta-analysis was not performed due to substantial methodological and measurement variability across studies.

### 2.10. Reporting Bias Assessment

We attempted to mitigate publication bias by searching multiple databases spanning biomedical, nursing/allied health, and interdisciplinary literature and by screening reference lists of included studies. We restricted inclusion to English-language, full-text articles, which may introduce language and availability bias; this was recorded as a limitation.

## 3. Results

**Figure 1:**
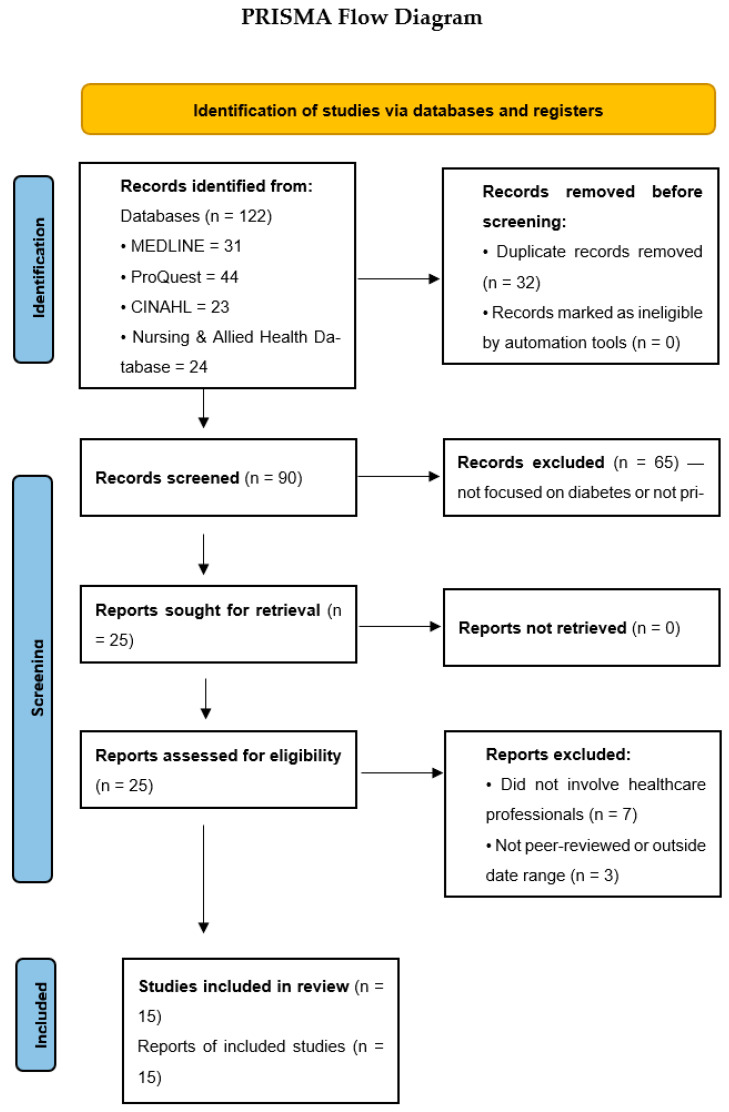
PRISMA flowchart with the search strategy of the systematic review.

This study followed the PRISMA guideline, providing guidance for conducting systematic reviews and other types of synthesis (Figure 1).

### 3.1. Study Selection

The search yielded 122 records on cultural competence in diabetes care. After de-duplication, 90 unique records remained. Following title/abstract screening and full-text assessment against the inclusion criteria, 15 studies were included.

Although this number may appear limited, it reflects the narrow scope of available research specifically addressing healthcare professionals’ cultural competence in diabetes care. Many of the excluded articles discussed cultural competence in general healthcare settings or other chronic conditions but did not focus on diabetes. Consequently, only studies that directly examined cultural competence among healthcare providers managing patients with diabetes were included to ensure the relevance and rigor of the synthesis. To ensure methodological rigor and the use of peer-reviewed evidence, only published studies in academic journals were included. Gray literature was excluded due to the lack of standardized peer review and variable methodological reporting.

### 3.2. Study Characteristics

The 15 included studies (Appendix A), conducted across multiple settings, assessed healthcare professionals’ cultural competence and the barriers they encounter when caring for minority populations with diabetes; several also evaluated educational programs to enhance competence. Five used cross-sectional surveys [16,17,18,19,20]; three employed case–control studies [12,21,22]; one was quasi-experimental [23]; five used qualitative methods such as semi-structured interviews or talking circles to explore provider and patient perspectives [24,25,26,27,28]; and one was a randomized controlled trial [13].

### 3.3. Risk of Bias in Studies

The Joanna Briggs Institute (JBI) Qualitative Assessment and Review Instrument Critical Appraisal Checklist was employed to appraise the quality of the articles included in this review. This instrument evaluates methodological rigor and identifies potential sources of bias in study design, execution, and analysis. All studies met the JBI criteria, providing comprehensive and detailed accounts of their methodologies and procedures (Table 3, Table 4, Table 5, Table 6 and Table 7). The JBI checklists, which include the full wording of all appraisal questions, are presented in Appendix B.

### 3.4. Result of Individual Studies

The synthesis included 15 studies published between 2015 and 2025, conducted across diverse settings including the UK, Sweden, Turkey, Germany, Canada, Australia, and East Asia. Sample sizes ranged from small qualitative groups (*n* = 12 dieticians) to large quantitative surveys (*n* = 672 healthcare professionals). Participants included nurses, dieticians, general practitioners, pharmacists, public health nurses, and other allied professionals.

### 3.5. Results of Synthesis

This review identified three themes: (1) healthcare professionals’ cultural competence in diabetes care, (2) barriers to delivering culturally competent diabetes care, and (3) effectiveness of cultural-competence training.

#### 3.5.1. Healthcare Professionals’ Cultural Competence in Diabetes Care

The evidence shows that healthcare professionals working in diabetes care generally demonstrate moderate to high levels of cultural competence, though important gaps remain, particularly in communication and organizational support.

Çınar et al. (2022) [18] evaluated 183 diabetes nurses using the Nurse Cultural Competence Scale (NCCS-T). The median score was 72, with nearly 90% scoring above average (≥60). All nurses scored above threshold levels in knowledge, skills, and sensitivity, yet language (63.9%) and communication (60.1%) difficulties were common [18]. Although most relied on informal sources such as family (85.8%) and social media (78.7%) for cultural knowledge, 88.5% nonetheless felt competent in caring for diverse patients. Higher competence was associated with graduate education, longer professional experience, and prior cultural training. Similar findings emerged in the study by Pettersson et al. (2020) [17], which assessed 279 diabetes care professionals using the Cultural Competence Assessment Instrument (CCAI-S). Most respondents reported acquiring competence through practice (78%) or self-directed learning (37%), with only 21% citing formal education [17]. Openness and awareness scored highest (mean 4.98/6), followed by interaction skills (4.40), whereas workplace support was rated lowest (3.30). While 58% demonstrated good openness and awareness, only 6% reported strong workplace support, highlighting weak institutional backing despite individual motivation.

At the service level, Zeh et al. (2015) [16] found notable variability across 34 diabetes care institutions. Using the Culturally Competent Assessment Tool (CCAT), 56% of services were rated highly competent (90–100%), 26% moderately competent (70–89%), and 18% below competent (<70%) [16]. Most organizations offered at least one culturally adapted element, such as language-specific consultations or tailored dietary advice, though one facility reported none.

Taken together, these studies suggest that while individual professionals often report strong openness, awareness, and self-perceived competence, structural and organizational supports are limited, and communication barriers remain widespread. The evidence points to a need for greater institutional commitment and system-level strategies to strengthen culturally competent diabetes care.

#### 3.5.2. Barriers to Providing Culturally Competent Diabetes Care

Several studies highlighted the multiple and intersecting barriers that limit culturally competent diabetes care, spanning linguistic, cultural, organizational, and structural domains. Jager et al. (2020) [25] examined dietitians’ experiences of working with migrant patients through semi-structured interviews. Dietitians consistently described language barriers as the most significant obstacle, disrupting information exchange, explanation of diet–diabetes links, and the establishment of trust [25]. Many reported low adherence to dietary advice, missed appointments, and incomplete food diaries among migrant patients. Although all participants were committed to providing high-quality care, a substantial proportion expressed dissatisfaction and insecurity in these consultations, citing limited cultural understanding. The authors concluded that structured cultural competence training, particularly in cultural knowledge, was urgently needed. At the service level, Zeh et al. (2015) almost all facilities reported barriers included language differences, entrenched food traditions, and cultural gaps between providers and patients [16]. Organizational challenges, low staffing, inadequate funding, and limited training were also widely cited. Only five of the 34 facilities reported encountering no cultural challenges in routine care.

Ofosu et al. (2023) explored providers’ perspectives through four focus groups and nine interviews with health professionals working with immigrant and refugee patients [24]. Trust-building was identified as a central challenge, often hindered by language barriers, low literacy, and patients’ unfamiliarity with the healthcare system. Some patients viewed care as transactional or acute, rather than preventive or relational, complicating long-term management. Non-medical burdens—socioeconomic stress, trauma, housing insecurity, and childcare, frequently disrupted diabetes management, particularly within time-limited consultations. Providers called for multilingual staff and culturally sensitive communication but acknowledged significant constraints in resources, training, and system-level coordination, especially for Indigenous populations.

Barriers reported by Indigenous patients, caregivers, and healthcare professionals highlighted additional layers of inequity. Tremblay et al. (2020) identified racism, differential treatment, neglect of socioeconomic realities, and medical jargon as key obstacles reported by patients and caregivers [26]. Healthcare professionals pointed to colonialism-related mistrust, difficulties building rapport, overcrowded systems that limited patient-centered approaches, and a lack of understanding of traditional and spiritual practices. Similarly, Crowshoe et al. (2018), in interviews with 28 physicians, described frustration with resource inequities, inconsistent services, and workforce shortages that undermined trust and continuity of care [27]. Clinicians also criticized colleagues for unrealistic expectations, limited cultural safety, bias, excessive reliance on guidelines, and overuse of medical jargon. While physicians emphasized the value of cultural and community supports—such as traditional food gatherings, poverty, stress, and historical trauma were consistently identified as structural barriers to effective diabetes care.

Together, these findings illustrate that while many professionals are motivated to provide culturally sensitive care, systemic barriers, particularly language and health literacy gaps, organizational constraints, and deep-rooted inequities, continue to limit its effective delivery.

#### 3.5.3. Effectiveness of Trainings on Cultural Competence for Healthcare Professionals

The studies reviewed consistently showed that cultural competence training improves healthcare providers’ knowledge, attitudes, confidence, and patient outcomes. However, the effects were often low and tended to diminish over time without reinforcement or systemic support.

Several interventions demonstrated short-term gains in knowledge and preparedness. For example, Jager et al. (2024) reported improved attitudes (83% to 92%) and consultation competence (60% to 80%) among dietitians following tailored training, though skills and some measures declined by two months [19]. Similarly, Lin and Hsu (2020) found small but significant increases in cultural competence scores among nurses after a 12 h program [13], while Rissel et al. (2022) observed improved cultural safety attitudes immediately post-training, which fell below baseline at follow-up [12]. Beck et al. (2024) also noted improved knowledge and awareness after a two-day course, but enthusiasm and behavioral skills decreased over time, highlighting the challenge of sustaining practice change [23].

Training sometimes translated into better patient outcomes. Movafagh and Adam (2024) showed that pharmacist-led training reduced provider unpreparedness and was associated with improved diabetes control among Asian patients, with mean HbA1c decreasing from 6.9 to 6.7 overall and from 8.0 to 7.65 in poorly controlled cases [20]. Kim and Lee (2016) demonstrated that public health nurses who underwent a four-week program achieved higher transcultural self-efficacy, stronger nurse–client trust, and greater satisfaction among migrant women compared with controls [21].

Large-scale and multicomponent programs appeared particularly impactful. McElfish et al. (2017) reported that across 51 sessions delivered to 1250 healthcare professionals, 91% of participants reported increased knowledge, 87% greater competence, and 87% improved performance [22]. Importantly, these training courses also prompted organizational changes, such as hiring diverse staff and embedding cultural training into practice.

Qualitative insights reinforced these findings. Nurses described greater awareness of their own biases and improved confidence in delivering individualized care, though concerns remained about adequately accommodating religious customs [28]. Feedback across studies emphasized the value of practical strategies such as teach-back, negotiation of treatment options, and efforts to use patients’ home languages.

Overall, cultural competence training reliably improves proximal outcomes and, in some cases, clinical indicators. Yet, sustaining these gains requires reinforcement, supervision, and organizational change. The evidence suggests that durable improvements are most likely when individual training is integrated with system-level supports and community-informed approaches.

### 3.6. Reporting Biases

Risk of bias was generally low to moderate. Studies using validated cultural competence tools (e.g., NCCS, TSET, CCCHP, CCAI-S) demonstrated stronger methodological rigor than those relying on self-report interviews alone. Several studies faced limitations such as small sample sizes, low follow-up rates, or potential response bias in self-perceived competence measures.

### 3.7. Certainty of Evidence

Using the GRADE framework, the overall certainty of evidence across outcomes ranged from low to moderate. Evidence supporting improvements in healthcare professionals’ knowledge, attitudes, and confidence following cultural competence training was rated as moderate, reflecting consistent findings across several study designs. In contrast, evidence related to skill application, patient outcomes, and organizational change was rated as low due to methodological limitations, small sample sizes, and variability between studies. Overall, the findings suggest promising effects of cultural competence training, but further high-quality research is needed to strengthen the evidence base in diabetes care contexts.

## 4. Discussion

This systematic review synthesized evidence on healthcare professionals’ cultural competence in diabetes care, barriers to its implementation, and the effectiveness of training interventions. Across studies, healthcare providers generally demonstrated moderate levels of cultural competence, though notable gaps persisted, particularly in communication, workplace support, and structural enablers of culturally safe practice.

Consistent with earlier research [29,30], three interrelated categories of barriers were identified: patient-related (e.g., language and health literacy challenges, cultural differences in perceptions of care), clinical-related (e.g., limited knowledge of migrants’ traditions, reliance on medical jargon, low adherence), and system-related (e.g., staff shortages, fragmented services, and limited institutional commitment). Studies involving Indigenous communities further highlighted the compounding effects of racism, historical trauma, and colonialism-related mistrust [26,27]. These findings underscore that cultural competence is not solely an individual skillset but is shaped and constrained by broader structural determinants.

Communication emerged as the most frequently reported obstacle, echoing earlier findings [31]. Language barriers not only impeded mutual understanding but also undermined trust and complicated patient education on treatment options. Migrants often faced the additional challenge of limited proficiency in the local language [32]. While some organizations attempted to address these issues through bilingual staff or interpreters [16], many healthcare professionals still reported insecurity during consultations, difficulty discussing sensitive topics, and reliance on informal cultural knowledge, such as family or social networks, rather than structured, evidence-based resources [18]. The persistence of these barriers underscores the need to systematically integrate linguistic and cultural mediation into diabetes care.

Socioeconomic factors also proved critical, with poverty, stress, housing insecurity, and competing life demands frequently limiting patients’ capacity for diabetes self-management [20]. These findings reinforce that cultural competence cannot be divorced from the social determinants of health. As highlighted by Powell (2016), cultural competence is essential for reducing health disparities but insufficient on its own; eliminating inequities also requires attention to broader structural barriers such as socioeconomic disadvantage, systemic inequities, and contextual health determinants [32,33]. Providers who focus narrowly on clinical guidance without acknowledging these realities risk further eroding patient trust.

The review also found that healthcare professionals often equated cultural competence with awareness and sensitivity, yet demonstrated more limited cultural knowledge and practical skills. This imbalance mirrors earlier studies showing that while providers may recognize cultural differences, they often lack the tools to adapt care [34]. Greater competence was associated with graduate education, longer clinical experience, and prior cultural training [17,18], suggesting that competence develops through both formal and experiential learning. However, workplace support remained weak, with only a minority of professionals reporting institutional backing for culturally competent practice [17].

Cultural competence training demonstrated clear benefits, with most interventions reporting improvements in knowledge, awareness, attitudes, and confidence [13,21,22]. Some programs also translated into measurable patient gains, including reduced HbA1c levels [20] and enhanced patient satisfaction and trust [21]. Nonetheless, effects were often modest and waned without reinforcement, underscoring the need for sustained, longitudinal approaches [12,23]. As suggested by Jettner et al. (2025), future efforts should move beyond brief didactics toward structured, interdisciplinary, community-engaged models grounded in cultural humility [35]. Examples include service-learning formats that embed critical reflection, provide reciprocal benefit for host communities, and encourage interprofessional teamwork, with outcomes tracked over time. Such designs are more likely to convert short-term gains into durable practice change and patient benefit. Qualitative studies further highlighted gaps in addressing specific needs, such as religious practices, and the risk of overly generic training [28].

Collectively, the evidence suggests that training alone is insufficient unless supported by systemic change. Durable improvements are most likely when education is embedded within organizational structures, reinforced through ongoing supervision, and co-designed with communities to ensure relevance. Structural measures such as hiring multilingual staff, developing culturally adapted educational materials, and addressing socioeconomic determinants are equally critical for advancing equity in diabetes care. Taken together, these findings highlight that advancing cultural competence in diabetes care requires both individual training and systemic transformation.

This review also highlights gaps in the literature. Despite the growing interest in cultural competence, research specifically addressing its role in diabetes care remains scarce, leaving critical gaps in understanding how best to support culturally diverse patients with this chronic condition. Additionally, many studies relied on self-reported measures of competence, which may be subject to social desirability bias, and most captured provider perspectives only. Few incorporated patient perspectives, with exceptions in Indigenous-focused research [26,27]. Furthermore, small samples and region-specific contexts limit generalisability. There remains a need for larger, longitudinal studies using validated and standardized tools, combined with robust patient-reported outcomes, to clarify which training models and institutional strategies are most effective in different contexts. In sum, while healthcare providers often demonstrate motivation and baseline awareness, communication challenges, structural inequities, and inadequate system-level support continue to constrain culturally competent diabetes care. Training interventions show promise but require reinforcement and integration with broader organizational and policy-level strategies. Future research should prioritize multi-level interventions that address both provider skills and the systemic determinants shaping diabetes outcomes in culturally diverse populations.

Implications for healthcare practice and education:

The hyper-diversity of migrant and ethnic minority populations makes it impossible for healthcare professionals to know every culture; however, cultural competence training is vital to help them recognize and address unconscious biases that may influence care delivery. Training should include practical examples of how cultural beliefs shape diet, physical activity, and health behaviors, along with tools such as teach-back methods and culturally appropriate communication strategies. Regular assessments using validated instruments like the CCAI or NCCS, together with patient feedback, can guide continuous improvement and adaptation. Healthcare organizations should foster inclusive environments by employing diverse staff, offering bilingual materials, and addressing social determinants that affect migrants. Moreover, cultural competence education must be ongoing, integrated into both undergraduate curricula and professional development, using interactive approaches such as role-play and case-based discussions to ensure sustained, patient-centered, and equitable diabetes care.

## 5. Conclusions

This review highlights the importance of cultural competence in diabetes care, showing its potential to improve outcomes by addressing communication and cultural barriers. While training enhances providers’ knowledge, attitudes, and skills, challenges remain in primary care settings, especially with immigrant and refugee populations. Sustainable improvements require not only training but also institutional support and structural change to achieve equitable, culturally responsive care.

## Figures and Tables

**Table 1:** Inclusion and Exclusion Criteria.

Inclusion Criteria	Exclusion Criteria
Articles published within the last ten years	Articles published more than ten years ago
Studies published in English	Studies issued in languages other than English
Research involving any health professionals	Research involving no health professional
Full-text articles that are available for review and analysis	Abstract-only publications or publications without access

**Table 2:** The database-specific EBSCOhost search strings.

Population	Interest	Context
(“healthcare professionals” OR “health workers” OR “nurses” OR “doctors” OR “clinicians” OR “medical staff”)	(“training” OR “education” OR “intervention” OR “program”) AND (“cultural competence” OR “cultural sensitivity” OR “cultural awareness” OR “cross-culture”)	(“diabetes” OR “diabetes mellitus” OR “diabetic care”)

**Table 3:** JBI Critical Appraisal Checklist for Analytical Cross-sectional studies.

Authors and Year	Q1	Q2	Q3	Q4	Q5	Q6	Q7	Q8
Zeh, P., Cannaby, A.-M., Sandhu, H.K., Warwick, J. and Sturt, J.A. (2018) [16]	Yes	Yes	Yes	Yes	Unclear	Yes	Yes	Yes
ÇINAR, D., OLGUN, N. and KOYUNCU, A. (2022) [18]	Yes	Yes	Yes	Yes	Yes	Yes	Yes	Yes
Pettersson, S., Holstein, J., Jirwe, M., Jaarsma, T. and Klompstra, L. (2022) [17]	Yes	Yes	Yes	Yes	Yes	Yes	No	Yes
Jager, M., Leij-Halfwerk, S., Reinier Akkermans, Rob and Maria (2024) [19]	Yes	Yes	Yes	Yes	Unclear	Yes	Yes	Yes
Movafagh, S and Adams. E (2024) [20]	Yes	Yes	Yes	Yes	Yes	Yes	No	Yes

**Table 4:** JBI Checklist for Case–Control Studies.

Authors and Year	Q1	Q2	Q3	Q4	Q5	Q6	Q7	Q8	Q9	Q10
McElfish, P.A, Long, C.R, Rowland, B, Moore, S, R. Wilmoth, and B. Ayers (2017) [22]	Yes	Yes	Yes	Yes	Yes	Yes	Unclear	Yes	Yes	Yes
Rissel, L. Liddle, C. Ryder, A. Wilson, M. Bower, and B. Richards (2022) [12]	Yes	Yes	Yes	Yes	Yes	Yes	Yes	Yes	Yes	Yes
Kim and H. Lee (2016) [21]	Yes	Yes	Yes	Yes	Yes	Yes	Unclear	Yes	Yes	Yes

**Table 5:** JBI Checklist for Quasi Experimental.

Authors and Year	Q1	Q2	Q3	Q4	Q5	Q6	Q7	Q8	Q9
Beck P et al. (2024) [23]	Yes	Yes	Yes	Yes	Yes	Yes	Yes	NA	Yes

**Table 6:** JBI Checklist for Randomized Controlled Trials.

Authors and Year	Q1	Q2	Q3	Q4	Q5	Q6	Q7	Q8	Q9	Q10	Q11	Q12	Q13
Lin M and Hsu H, (2020) [13]	Yes	Yes	Yes	Yes	Yes	Yes	Yes	NA	Yes	Yes	Yes	Yes	Unclear

**Table 7:** Risk of bias assessed by the Joanna Briggs Institute Critical Appraisal Checklist for Qualitative Research.

Authors and Year	Q1	Q2	Q3	Q4	Q5	Q6	Q7	Q8	Q9	Q10
N. Ofosu, T. Luig, N. Mumtaz, Y. Chiu, K. Lee, R.O Yeung, and D.L. Campbell-Schere (2023) [24]	Yes	Yes	Yes	Yes	Yes	Unclear	Yes	Yes	Yes	Yes
Jager M, den Boeft A, Leij-Halfwerk S, van der Sande R, van den Muijsenbergh M (2020) [25]	Yes	Yes	Yes	Yes	Yes	Yes	Yes	Yes	Yes	Yes
Tremblay, M., Bradette-Laplante, M., Witteman, H.O., Dogba, M.J., Breault, P., Paquette, J., Careau, E. and Echaquan, S. (2020) [26]	Yes	Yes	Yes	Yes	Yes	Yes	Yes	Yes	Yes	Yes
Kaihlanen, A.-M., Hietapakka, L. and Heponiemi, T. (2019) [28]	Yes	Yes	Yes	Yes	Yes	Unclear	Yes	Yes	Yes	Yes
Crowshoe, L., Henderson, R.I., Green, M.E., Jacklin, K.M., Walker, L.M. and Calam, B. (2018) [27]	Yes	Yes	Yes	Yes	Yes	Yes	Yes	Yes	Yes	Yes

## Data Availability

All data supporting this systematic review are derived from previously published studies and datasets, as cited in the manuscript. Processed data are presented in Appendix A and in the reference list. Additional details are available from the corresponding author upon reasonable request.

## Appendix A

**Table A1:** Study Characteristics and Key Findings.

Authors/Date	Aim	Methodology	Sample	Findings
Zeh et al. (2018) [16]	This study aims to assess the cultural competence of diabetes services provided to minority ethnic groups in a multicultural UK metropolis with a diabetes prevalence of 4.3%	The culturally competent assessment tool (CCAT) was used to evaluate the cultural competence of diabetes services.	The survey was sent to each senior general practitioner (GP) and practice manager of 66 general practices, but only 34 practices responded.	An assessment of cultural competence using CCAT revealed that 19 practices were highly culturally competent (90–100%), 9 moderately competent (70–89%), and 6 less competent (<70%)
Pettersson S et al. (2022) [17]	This study evaluates the cultural competence of Swedish healthcare professionals specialized in diabetes care and identifies factors influencing it.	The Swedish version of the Cultural Competence Assessment Instrument (CCAI-S) was employed to assess cultural competence. The instrument consists of 13 items, with responses measured on a 6-point scale.	279 healthcare professionals responded to the questionnaire. The healthcare professionals consisted of 207 registered nurses, 37 general practitioners, 22 podiatrists, 8 dieticians, and 4 other professionals.	Overall, 61% felt confident in verbal communication across cultures, whereas 9% struggled with non-verbal communication and 10% with minority ethnic clients.
Çinar et al. (2022) [18]	The study investigates diabetes nurses’ cultural competence in educating and caring for patients with diabetes, taking into account their cultural characteristics.	A cross-sectional descriptive study was carried out, and data were obtained by using the introductory information Form and the Nurse Cultural Competence Scale (NCCS-T)	The study consisted of 183 diabetes nurses in Turkey	63.9% of diabetes nurses identified language as a problem, and 60.15% had communication issues. According to the NCCS-T scores, all participants had cultural skills, knowledge, and sensitivity. 89.65% had an average level of cultural competence and a higher mean score in graduate and experienced nurses.
Ofosu et al. (2023) [24]	The study was carried out to understand primary health providers’ views on the challenges of providing intercultural diabetes and obesity care.	This research included 9 individual interviews and 4 focus groups with 6 to 12 participants. Semi-structured and open-ended questions that address how healthcare providers manage diabetes and obesity and the challenges they experience were used in the interview.	This study involved 37 healthcare professionals specialized in diabetes care.	Communication barriers between patients and providers, low literacy levels of migrants, and a lack of knowledge in addressing non-medical issues affected the health outcomes of migrant patients.
Jager M et al. (2020) [25]	to investigate dieticians’ experiences, knowledge, Attitudes, and abilities they consider crucial for effective dietetic diabetes care	A semi-structured interview was designed using Seeleman’s cultural competency model and the Dutch dietetic consultation model.	12 dieticians of different ages, ethnic backgrounds and experience were involved	Dietitians were dissatisfied with their consultations with immigrants due to communication issues like difficulty in retrieving information and explaining how food and diabetes work.
Tremblay M et al. (2020) [26]	Aimed to find potential obstacles and facilitators of cultural safety in medical care provided to Indigenous people with diabetes.	Using a qualitative descriptive design, data were collected through three talking circles with three different groups of participants.	A total of 30 participants, including 10 healthcare professionals, 6 caregivers, and 14 people with diabetes	Health providers mentioned the congestion in the healthcare system, communication barriers, and lack of knowledge about the culture as hindrances to culturally safe care.
Crowshoe L et al. (2018) [27]	Aimed to describe the perspectives of Canadian physicians on diabetes management for Indigenous patients.	A semi-structured interview involving family physicians, specialists, and Aboriginal patients was carried out.	28 individuals took part in this study.	Physicians acknowledged their limitations, linking them to inconsistent service, physician shortage, lack of trust, and discrimination by other health professionals.
Jager M et al. (2024) [19]	The purpose of this study was to present the pilot implementation and initial evaluation of a cultural competence program for dieticians.	Training was created based on Seeleman’s cultural competence framework. Evaluation was conducted using a Cultural Competence Questionnaire, an Experience Evaluation Questionnaire, and consultation observations.	8 dieticians responded and participated in the research	There was an increase in self-perceived cultural competence and attitude, the use of teach-back methods and discussions of treatment options. Skills and knowledge were unchanged.
Movafagh S and Adams E (2024) [20]	This paper assesses the effect of pharmacist-led cultural competency training on provider self-perceived readiness and diabetes-related health outcomes in Asian patients.	A cross-sectional study, followed by a quasi-experimental Phase 1, evaluated the relationship between patients’ diabetes health and physicians’ CCCS scores. In phase two, surveys and patients’ diabetes indicators before and after training were used to assess pharmacist-led cultural competency training.	The first phase involved 9 healthcare providers. The second phase included 30 providers and 95 patients	Phase 1 demonstrated that providers’ baseline cross-cultural competence was insufficient, whereas Phase 2 found that training reduced their perceived unpreparedness. Only patients with uncontrolled diabetes showed a significant reduction in health parameters.
Kim K and Lee H (2016) [21]	This study used intervention mapping to assess the impact of a cultural competence training program for public health nurses (PHNs).	An embedded mixed-methods design was adopted, with professionals divided into experimental and control groups. The experimental group had four weeks of cultural competence training. Cultural competence was measured using the Koreans’ version of Jeffrey’s Transcultural Self-efficacy Tool (TSET). It also featured migrant women receiving treatment.	41 public health nurses and 40 migrant women participated	The experimental group significantly improved Transcultural Self-efficacy (TSE), client-nurse trust, and satisfaction with nursing care compared to the control group.
Beck P et al. (2024) [23]	The study’s objective is to assess the efficacy of cross-cultural competence training for German healthcare professionals.	A quasi-experimental evaluation study was carried out at two German hospitals. The self-reported questionnaire Cross-Cultural Competence of Healthcare Professionals (CCCHP) was used to assess cross-cultural competence in both an intervention and control group, and the results were analyzed using SPSS Statistics 25.	196 healthcare providers contributed to this research	Cross-cultural training increased knowledge, awareness, and attitudes while decreasing social desirability. Motivation and curiosity decreased, although empathy increased marginally. However, cross-cultural skills decreased after training, emphasizing the need for additional practical, long-term training.
Lin M and Hsu H (2020) [13]	This study aimed to examine the impact of a cultural competency educational course on nurses’ self-assessments of their cultural competence.	Attendees were randomly assigned to one of two groups: experimental or control. A four-week course was held, and pre-post data were collected with a two-month follow-up. Cultural competency was measured using the NCCS.	A total of 97 nurses were included	The experimental group scored lower on the total cultural competence and cultural action ability before intervention; however, the score was higher than that of the control group after a two-month follow-up.
Rissel C et al. (2022) [12]	This study aimed to assess the effectiveness of a Central Australian cultural awareness training program for healthcare professionals.	A case–control study was employed with a consecutive group pre-program, post-program, and follow-up evaluation.	Only 11 out of 124 participants responded to the two-month follow-up.	The scale mean score pre-program was 45.7, 47.3 post-program, and 42.2 after a two-month follow-up. Most participants found the course relevant to their work, and the qualitative feedback was positive.
McElfish P et al. (2017) [22]	The purpose of this study was to develop and evaluate a cultural training program to improve the delivery of culturally appropriate care in indigenous communities.	A mixed-methods evaluation based on the Kirkpatrick model was employed, with quantitative data collected after each session and qualitative data acquired from individuals and organizational units at two time points: immediately after each session and six months later.	672 healthcare professionals participated in the research	Following training, 91.2% indicated more knowledge, 86.6% higher competence, and 87.2% better performance. At six months, the response rates were 17% for individuals and 28% for organizations.
Kaihlanen AM et al. (2019) [28]	This study investigates the nurses’ perceptions of the content and utility of cultural competence training, which focuses on developing awareness of one’s cultural differences.	A semi-structured interview was held in a hospital with a large number of immigrants. Invitations for cultural competence training and post-interview were sent via email and through the ward managers.	Only 20 registered nurses and practical nurses indicated interest, but only 10 attended the two sessions	Nurses acknowledged that the training raises understanding of patients’ cultural diversity, which fosters respect in treatment. It also promotes self-awareness and cognitive shifts and justifies using effective communication strategies.

## Appendix B

**Table A2:** JBI Critical Appraisal Checklist for Analytical Cross Sectional Studies.

	Yes	No	Unclear	Not Applicable
Were the criteria for inclusion in the sample clearly defined?	□	□	□	□
2. Were the study subjects and the setting described in detail?	□	□	□	□
3. Was the exposure measured in a valid and reliable way?	□	□	□	□
4. Were objective, standard criteria used for measurement of the condition?	□	□	□	□
5. Were confounding factors identified?	□	□	□	□
6. Were strategies to deal with confounding factors stated?	□	□	□	□
7. Were the outcomes measured in a valid and reliable way?	□	□	□	□
8. Was appropriate statistical analysis used?	□	□	□	□

## References

[1] Hossain M.J., Al-Mamun M., Islam M.R. (2024). Diabetes mellitus, the fastest growing global public health concern: Early detection should be focused. Health Sci. Rep..

[2] World Health Organization (2024). Diabetes.

[3] Yapislar H., Gurler E.B. (2024). Management of microcomplications of diabetes mellitus: Challenges, current trends, and future perspectives in treatment. Biomedicines.

[4] Ahmad F.J., Joshi S.H. (2023). Self-care practices and their role in the control of diabetes: A narrative review. Cureus.

[5] International Organization for Migration (2024). World Migration Report 2024.

[6] Theodosopoulos L., Fradelos E.C., Panagiotou A., Dreliozi A., Tzavella F. (2024). Delivering culturally competent care to migrants by healthcare personnel: A crucial aspect of culturally sensitive care. J. Nurs. Health Sci..

[7] Stubbe D.E. (2020). Practicing cultural competence and cultural humility in the care of diverse patients. Focus (Am. Phychiatr. Publ.).

[8] Hassan S., Gujral U.P., Quarells R.C., Rhodes E.C., Shah M.K., Obi J., Lee W.-H., Shamambo L., Weber M.B., Narayan K.M.V. (2023). Disparities in diabetes prevalence and management by race and ethnicity in the USA: Defining a path forward. Lancet Diabetes Endocrinol..

[9] Taylor Y.J., Spencer M.D., Mahabaleshwarkar R., Ludden T. (2017). Racial/ethnic differences in healthcare use among patients with uncontrolled and controlled diabetes. Ethn. Health.

[10] Cipta D.A., Andoko D., Theja A., Utama A.V.E., Hendrik H., William D.G., Reina N., Handoko M.T., Lumbuun N. (2024). Culturally sensitive patient-centered healthcare: A focus on health behavior modification in low- and middle-income nations—Insights from Indonesia. Front. Med..

[11] Rahimi M., Khodabandeh Shahraki S., Fatehi F., Farokhzadian J. (2023). A virtual training program for improving cultural competence among academic nurse educators. BMC Med. Educ..

[12] Rissel C., Liddle L., Ryder C., Wilson A., Bower M., Richards B. (2022). Impact evaluation of a Central Australian Aboriginal cultural awareness training program for health professionals and students. J. Aust. Indig. HealthInfoNet.

[13] Lin M.H., Hsu H.C. (2020). Effects of a cultural competence education programme on clinical nurses: A randomised controlled trial. Nurse Educ. Today.

[14] Munn Z., Peters M.D.J., Stern C., Tufanaru C., McArthur A., Aromataris E. (2018). Systematic review or scoping review? Guidance for authors when choosing between a systematic or scoping review approach. BMC Med. Res. Methodol..

[15] Page M.J., McKenzie J.E., Bossuyt P.M., Boutron I., Hoffmann T.C., Mulrow C.D., Shamseer L., Tetzlaff J.M., Akl E.A., Brennan S.E. (2021). The PRISMA 2020 statement: An updated guideline for reporting systematic reviews. BMJ.

[16] Zeh P., Cannaby A.M., Sandhu H.K., Warwick J., Sturt J.A. (2018). A cross-sectional survey of general practice health workers’ perceptions of their provision of culturally competent services to ethnic minority people with diabetes. Prim. Care Diabetes.

[17] Pettersson S., Holstein J., Jirwe M., Jaarsma T., Klompstra L. (2022). Cultural competence in healthcare professionals specialised in diabetes working in primary healthcare: A descriptive study. Health Soc. Care Community.

[18] Çınar D., Olgun N., Koyuncu A. (2022). Investigation of the cultural competence levels of diabetes nurses. Clin. Exp. Health Sci..

[19] Jager M., Leij-Halfwerk S., Akkermans R., Maria R. (2024). Cultural competence training of dietitians: Development and preliminary evaluation. Prim. Health Care Res. Dev..

[20] Movafagh A., Adams S. (2023). The impact of a pharmacist-led cultural competence training on provider perceived preparedness and clinical care in patients with diabetes of Asian descent. J. Am. Pharm. Assoc..

[21] Kim Y.K., Lee H. (2016). The effectiveness of a cultural competence training program for public health nurses using intervention mapping. J. Korean Acad. Community Health Nurs..

[22] McElfish P.A., Long C.R., Rowland B., Moore S., Wilmoth R., Ayers B. (2017). Improving culturally appropriate care using a community-based participatory research approach: Evaluation of a multicomponent cultural competency training program, Arkansas, 2015–2016. Prev. Chronic Dis..

[23] Beck P., Matusiewicz D., Schouler-Ocak M., Khan Z., Peppler L., Schenk L. (2024). Evaluation of cross-cultural competence among German healthcare professionals: A quasi-experimental study of training in two hospitals. Heliyon.

[24] Ofosu N.N., Luig T., Mumtaz N., Chiu Y., Lee K.K., Yeung R.O., Campbell-Scherer D.L. (2023). Health care providers’ perspectives on challenges and opportunities of intercultural health care in diabetes and obesity management: A qualitative study. Can. Med. Assoc. J. Open.

[25] Jager M., den Boeft A., Leij-Halfwerk S., van der Sande R., van den Muijsenbergh M. (2020). Cultural competency in dietetic diabetes care: A qualitative study of the dietitian’s perspective. Health Expect..

[26] Tremblay M., Bradette-Laplante M., Witteman H.O., Dogba M.J., Breault P., Paquette J., Careau E., Echaquan S. (2021). Providing culturally safe care to Indigenous people living with diabetes: Identifying barriers and enablers from different perspectives. Health Expect..

[27] Crowshoe L., Henderson R.I., Green M.E., Jacklin K.M., Walker L.M., Calam B. (2018). Exploring Canadian physicians’ experiences with type 2 diabetes care for adult Indigenous patients. Can. J. Diabetes.

[28] Kaihlanen A.M., Hietapakka L., Heponiemi T. (2019). Increasing cultural awareness: Qualitative study of nurses’ perceptions about cultural competence training. BMC Nurs..

[29] Bhattacharyya O.K., Estey E.A., Rasooly I.R., Harris S., Zwarenstein M., Barnsley J. (2011). Providers’ perceptions of barriers to the management of type 2 diabetes in remote Aboriginal settings. Int. J. Circumpolar Health.

[30] Caballero A.E. (2007). Cultural competence in diabetes mellitus care: An urgent need. Insulin.

[31] McClimens A., Brewster J., Lewis R. (2014). Recognising and respecting patients’ cultural diversity. Nurs. Stand..

[32] Khanal S.K. (2025). Language barriers and their consequences in healthcare: A qualitative case study of Nepali migrants in Finland. BMC Health Serv. Res..

[33] Powell D.L. (2016). Social determinants of health: Cultural competence is not enough. Creat. Nurs..

[34] Horvat L., Horey D., Romios P., Kis-Rigo J. (2014). Cultural competence education for health professionals. Cochrane Database Syst. Rev..

[35] Jettner J.F., Crawford K., Campbell A.D., Shackleford K., Bailey B. (2025). Long-term effect on cultural competency: University study abroad service learning course in Belize. J. Soc. Work. Educ..

